# Supplementary figures and images for: Codon optimization, expression in Escherichia coli, and immunogenicity analysis of deformed wing virus (DWV) structural protein
Source: PeerJ. 2020 Mar 11;8:e8750. doi: 10.7717/peerj.8750 (PMC7071823; doi:10.7717/peerj.8750)

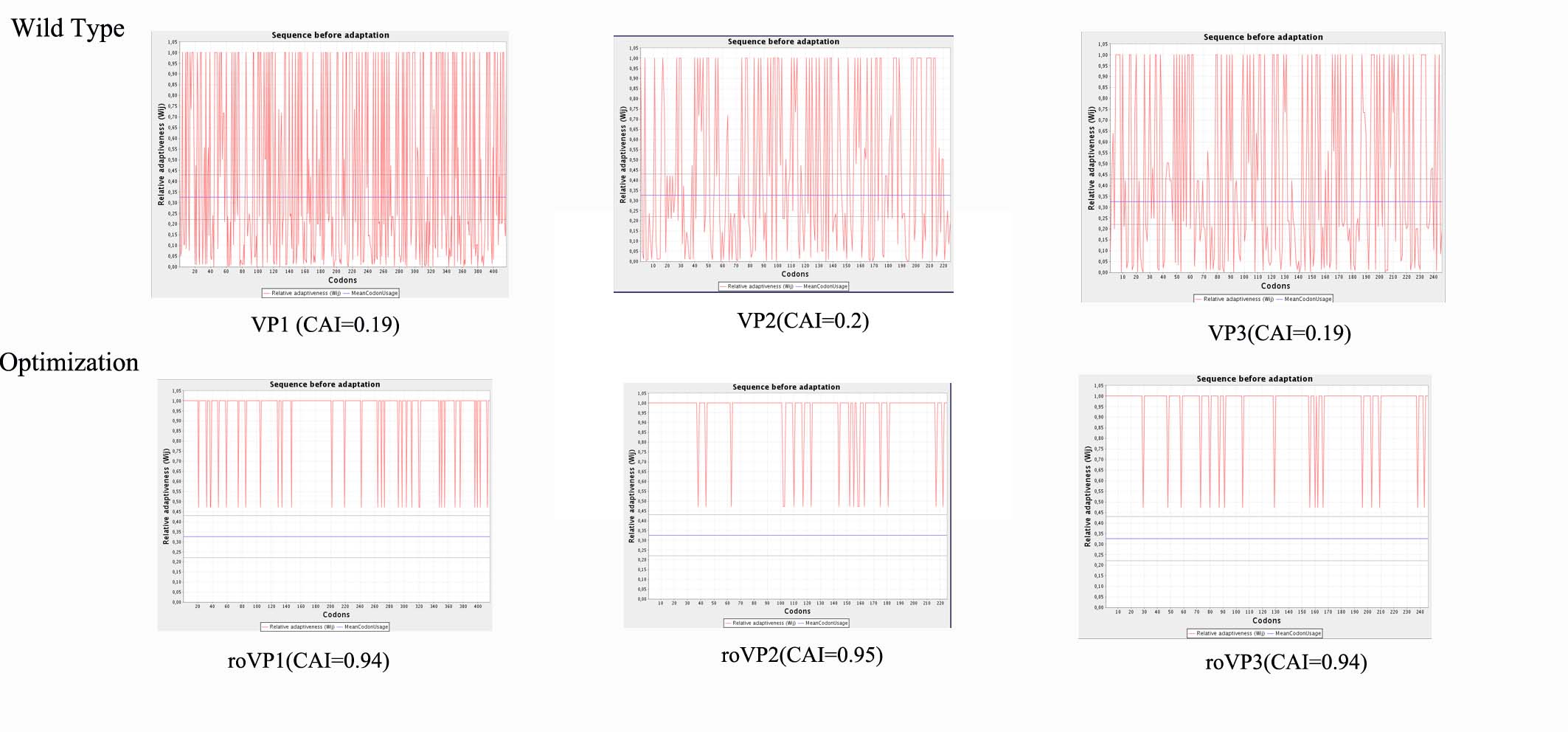

Supplement: Supplemental Information 1 [file peerj-08-8750-s001.jpg]

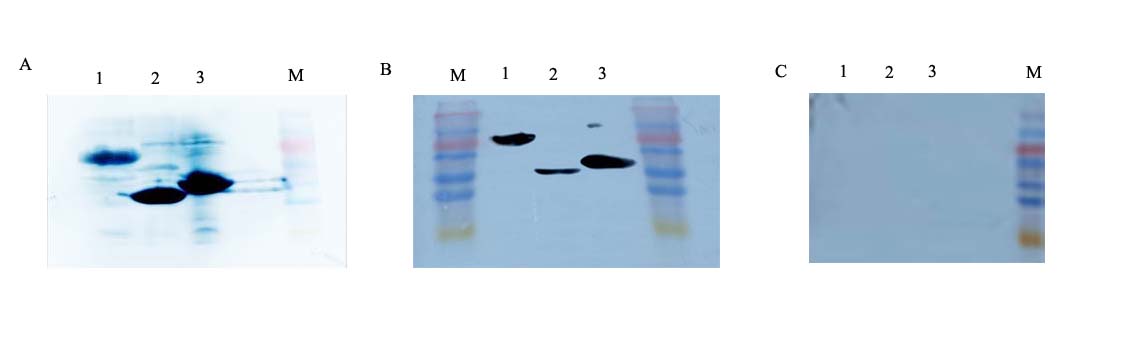

Supplement: Supplemental Information 2 — A, B and C represent the results of anti–DWV polyclonal antibody, anti–His tag antibody and healthy mice IgG as primary antibody, respectively. Lane 1; roVP1. Lane 2; roVP2. Lane 3; roVP3. M; Low molecular weight protein maker. [file peerj-08-8750-s002.jpg]
