# Supplementary material for: Codon optimization, expression in Escherichia coli, and immunogenicity analysis of deformed wing virus (DWV) structural protein
Source: PeerJ. 2020 Mar 11;8:e8750. doi: 10.7717/peerj.8750 (PMC7071823; doi:10.7717/peerj.8750)
Supplement: Supplemental Information 3 [file peerj-08-8750-s003.docx]

| **Codons** | **Wild-type** | | | **Optimized** | | | **Codons** | **Wild-type** | | | **Optimized** | | | **Codons** | **Wild-type** | | | **Optimized** | | | **Codons** | **Wild-type** | | | **Optimized** | | | |
| --- | --- | --- | --- | --- | --- | --- | --- | --- | --- | --- | --- | --- | --- | --- | --- | --- | --- | --- | --- | --- | --- | --- | --- | --- | --- | --- | --- | --- |
|  | **VP1** | **VP2** | **VP3** | **oVP1** | **oVP2** | **oVP3** |  | **VP1** | **VP2** | **VP3** | **oVP1** | **oVP2** | **oVP3** |  | **VP1** | **VP2** | **VP3** | **oVP1** | **oVP2** | **oVP3** |  | **VP1** | **VP2** | **VP3** | **oVP1** | **oVP2** | **oVP3** |  |
| **GCA(A)** | **9** | **5** | **5** | **0** | **0** | **0** | **GCC (A)** | **4** | **1** | **0** | **0** | **0** | **0** | **GCG (A)** | **4** | **5** | **3** | **33** | **18** | **18** | **GCT (A)** | **16** | **7** | **10** | **0** | **0** | **0** |  |
| **TGC (C)** | **1** | **0** | **0** | **2** | **5** | **2** | **TGT (C)** | **1** | **5** | **2** | **0** | **0** | **0** | **GAC (D)** | **4** | **2** | **1** | **19** | **12** | **16** | **GAT (D)** | **15** | **10** | **15** | **0** | **0** | **0** |  |
| **GAA (E)** | **13** | **5** | **6** | **18** | **6** | **12** | **GAG (E)** | **5** | **1** | **6** | **0** | **0** | **0** | **TTC (F)** | **4** | **1** | **1** | **19** | **9** | **8** | **TTT (F)** | **15** | **8** | **7** | **0** | **0** | **0** |  |
| **GGA (G)** | **9** | **5** | **1** | **0** | **0** | **0** | **GGC (G)** | **2** | **1** | **3** | **0** | **0** | **0** | **GGG (G)** | **0** | **3** | **1** | **0** | **0** | **0** | **GGT(G)** | **21** | **7** | **5** | **32** | **16** | **10** |  |
| **CAC (H)** | **2** | **1** | **1** | **10** | **5** | **4** | **CAT (H)** | **8** | **4** | **3** | **0** | **0** | **0** | **ATA (I)** | **4** | **4** | **4** | **0** | **0** | **0** | **ATC (I)** | **1** | **4** | **3** | **17** | **18** | **18** |  |
| **ATT (I)** | **12** | **10** | **11** | **0** | **0** | **0** | **AAA (K)** | **3** | **2** | **6** | **11** | **5** | **12** | **AAG (K)** | **8** | **3** | **6** | **0** | **0** | **0** | **TTA (L)** | **19** | **11** | **10** | **0** | **0** | **0** |  |
| **TTG (L)** | **8** | **2** | **5** | **0** | **0** | **0** | **CTA (L)** | **1** | **1** | **1** | **0** | **0** | **0** | **CTC (L)** | **0** | **0** | **0** | **0** | **0** | **0** | **CTG (L)** | **1** | **1** | **0** | **32** | **16** | **16** |  |
| **CTT (L)** | **3** | **1** | **0** | **0** | **0** | **0** | **ATG (M)** | **10** | **5** | **4** | **10** | **5** | **4** | **AAC (N)** | **0** | **2** | **2** | **17** | **12** | **13** | **AAT (N)** | **17** | **10** | **11** | **0** | **0** | **0** |  |
| **CCA (P)** | **11** | **6** | **6** | **0** | **0** | **0** | **CCC (P)** | **3** | **0** | **1** | **0** | **0** | **0** | **CCG (P)** | **3** | **2** | **3** | **29** | **12** | **15** | **CCT (P)** | **12** | **4** | **5** | **0** | **0** | **0** |  |
| **CAA (Q)** | **21** | **8** | **6** | **0** | **0** | **0** | **CAG (Q)** | **5** | **4** | **2** | **26** | **12** | **8** | **AGA (R)** | **3** | **4** | **4** | **0** | **0** | **0** | **AGG(R)** | **6** | **1** | **1** | **0** | **0** | **0** |  |
| **CGA (R)** | **3** | **2** | **3** | **0** | **0** | **0** | **CGC (R)** | **1** | **0** | **1** | **0** | **0** | **0** | **CGG (R)** | **1** | **1** | **1** | **0** | **0** | **0** | **CGT(R)** | **8** | **4** | **2** | **22** | **12** | **12** |  |
| **AGC (S)** | **2** | **2** | **4** | **0** | **0** | **0** | **AGT (S)** | **9** | **5** | **8** | **0** | **0** | **0** | **TCA (S)** | **7** | **0** | **2** | **0** | **0** | **0** | **TCC(S)** | **3** | **4** | **0** | **0** | **0** | **0** |  |
| **TCG (S)** | **3** | **1** | **4** | **0** | **0** | **0** | **TCT (S)** | **10** | **7** | **8** | **34** | **19** | **26** | **ACA (T)** | **6** | **1** | **4** | **0** | **0** | **0** | **ACC(T)** | **2** | **1** | **2** | **20** | **9** | **15** |  |
| **ACG (T)** | **5** | **3** | **1** | **0** | **0** | **0** | **ACT (T)** | **7** | **4** | **8** | **0** | **0** | **0** | **GTA (V)** | **13** | **6** | **4** | **0** | **0** | **0** | **GTC(V)** | **2** | **0** | **1** | **0** | **0** | **0** |  |
| **GTG (V)** | **7** | **2** | **6** | **0** | **0** | **0** | **GTT (V)** | **12** | **9** | **10** | **34** | **17** | **21** | **TGG (W)** | **12** | **3** | **7** | **12** | **3** | **7** | **TAC(Y)** | **2** | **0** | **0** | **19** | **14** | **9** |  |
| **TAT (Y)** | **17** | **14** | **9** | **0** | **0** | **0** | **TAA (.)** | **0** | **0** | **1** | **0** | **0** | **0** | **TGA (.)** | **0** | **0** | **0** | **0** | **0** | **0** | **TAG (.)** | **0** | **0** | **0** | **0** | **0** | **0** |  |

**Table 1S. Codon distribution of wild-type and optimized *VP1*, *VP2*, and *VP3* genes.**
